# Supplementary material for: Exploratory Quality Control Study for Polygonum multiflorum Thunb. Using Dinuclear Anthraquinones with Potential Hepatotoxicity
Source: Molecules. 2022 Oct 10;27(19):6760. doi: 10.3390/molecules27196760 (PMC9570674; doi:10.3390/molecules27196760)
Supplement: Supplementary file 1 [file molecules-27-06760-s001.zip › molecules-1935547-supplementary.pdf]

molecules

MDPI

Article

Exploratory Quality Control Study for *Polygonum multiflorum* Thunb. using Dinuclear Anthraquinones with potential hepatotoxicity

Huiyu Gao [1,†], Jianbo Yang [1,†], Xueting Wang [1], Yunfei Song [1,2], Xianlong Cheng [1], Feng Wei [1,*], Ying Wang [1], Donglin Gu [1], Hua Sun [3] and Shuangcheng Ma [1,*]

[1] Institute for Control of Chinese Traditional Medicine and Ethnic Medicine, National Institutes for Food and Drug Control, Beijing 102600, China
[2] School of Chinese Materia Medica, Beijing University of Chinese Medicine, Beijing, 102488, China
[3] Chinese Academy of Medical Sciences & Peking Union Medical College, Beijing 100050, China
* Correspondence: weifeng@nifdc.org.cn (F.W.); masc@nifdc.org.cn (S.M.); Tel.: +86-10-53852020 (F.W.); +86-10-53852076 (S.M.)
† These authors contributed equally to this work

Supplementary Materials

Table S1. Contents of free dianthrones in PMR.

Samples	Batch Number	Contents of analytes (μg/g)						
		1	2	3	4	5	6	Total△
PMR-01	AHBZ202001	0.2395	0.5175	0.0643	0.0467	0.0640	0.0424	0.9744
PMR-02	AHBZ202002	1.2660	0.5417	0.5121	0.4323	0.7091	0.3573	3.8185
PMR-03	AHBZ202003	1.0006	0.7852	0.9106	0.9739	2.6917	1.1963	7.5583
PMR-04	AHBZ202004	1.3388	1.1351	0.8042	1.1238	2.2179	1.2528	7.8726
PMR-05	AHBZ202005	2.6980	2.4985	2.1521	2.3733	5.9698	24.5818	40.2735
PMR-06	AHFY202001	0.4121	0.3304	0.1888	0.3053	0.6596	0.2784	2.1746
PMR-07	AHFY202002	0.8260	0.5518	0.7964	0.8730	2.5438	1.4055	6.9965
PMR-08	AHFY202003	0.7513	0.5388	0.9397	0.8948	3.6365	1.7461	8.5072
PMR-09	AHFY202004	0.9865	0.0886	0.4651	0.2463	0.8397	0.1518	2.778
PMR-10	AHFY202005	0.9639	0.1551	0.4534	0.2206	0.3169	0.1910	2.3009
PMR-11	GDGZ202002	72.0310	63.0322	59.2059	64.2005	53.0040	37.2258	348.6994
PMR-12	GDGZ202003	64.0366	61.9686	63.8661	67.3944	46.2105	31.5802	335.0564
PMR-13	GDGZ202004	64.5061	59.7736	56.2312	58.1959	75.0201	34.6500	348.3769
PMR-14	GDGZ202005	76.8091	71.7398	61.3272	63.6051	73.1711	33.7220	380.3743
PMR-15	SCDZ202001	0.2483	0.6101	0.3726	0.3818	0.2878	0.1355	2.0361
PMR-16	SCDZ202002	4.6900	3.6934	2.4969	2.5346	4.0790	1.6115	19.1054
PMR-17	SCDZ202003	14.3823	9.9849	11.2485	10.4674	19.7349	8.2563	74.0743
PMR-18	SCDZ202004	35.2265	28.5175	23.1835	22.9777	36.6123	16.0731	162.5906
PMR-19	SCDZ202005	9.2559	6.4017	2.6982	2.6204	2.2766	1.0394	24.2922
PMR-20	SCYB202001	8.7492	7.1704	6.1274	6.3185	13.1049	5.4630	46.9334
PMR-21	SCYB202002	15.1794	11.0861	10.3946	10.3586	20.1022	8.2738	75.3947
PMR-22	SCYB202003	7.2280	6.2994	5.6975	6.0441	13.1601	5.2547	43.6838
PMR-23	SCYB202004	7.1003	6.1151	5.3397	5.7406	11.9352	5.3909	41.6218
PMR-24	SCYB202005	13.1142	8.8357	7.1588	7.1281	10.6656	4.7937	51.6961
PMR-25	GZKL202001	4.4360	4.5713	3.1117	3.4860	7.2845	2.9515	25.841
PMR-26	GZKL202002	5.7612	5.6835	5.4832	5.8382	14.1750	5.6722	42.6133
PMR-27	GZKL202003	4.6648	5.1173	3.9051	4.4296	10.9197	4.0335	33.07
PMR-28	GZKL202004	8.4560	8.8157	7.0238	7.9926	18.3918	7.5759	58.2558

PMR-29	GZKL202005	8.7958	9.6978	5.8432	6.4548	12.5886	4.8962	48.2764
PMR-30	GZBJ202001	0.3710	0.3995	0.2666	0.3200	0.5866	0.2918	2.2355
PMR-31	GZBJ202002	0.1186	0.1278	0.1218	0.1440	0.4094	0.1663	1.0879
PMR-32	GZBJ202003	0.0813	0.0803	0.1049	0.1133	0.3133	0.1537	0.8468
PMR-33	GZBJ202004	0.8578	0.9532	0.5969	0.6553	1.2617	0.5594	4.8843
PMR-34	GZBJ202005	1.5167	1.7548	1.1213	1.2881	3.0908	1.3332	10.1049
PMR-35	GZAS202001	15.6055	17.5438	11.3977	13.3958	33.9562	14.1433	106.0423
PMR-36	GZAS202002	19.4390	22.0845	13.4459	14.9028	32.2931	14.0846	116.2499
PMR-37	GZAS202003	9.2096	10.1663	8.7225	9.7620	26.4248	11.2651	75.5503
PMR-38	GZAS202004	6.9156	7.4652	5.5873	6.4877	13.8430	5.7486	46.0474
PMR-39	GZAS202005	19.6010	21.9899	15.5834	17.8051	46.1957	19.1504	140.3255
PMR-40	GZTR202001	2.2621	2.6517	1.6851	1.9864	5.7646	2.2716	16.6215
PMR-41	GZTR202002	2.6692	3.1115	1.9317	2.1731	5.7187	2.4296	18.0338
PMR-42	GZTR202003	1.6258	1.8518	1.4298	1.5947	4.4652	1.8562	12.8235
PMR-43	GZTR202004	2.6159	3.0663	2.0064	2.0209	6.2378	13.8484	29.7957
PMR-44	GZTR202005	0.1463	0.9422	0.1948	0.2265	0.8003	0.3167	2.6268
PMR-45	YNHH20201101	10.1722	9.1809	7.4501	7.8558	17.5398	8.0480	60.2468
PMR-46	YNWS202002	4.9153	3.5966	2.9336	2.8624	7.7683	3.3382	25.4144
PMR-47	YNWS202003	7.6960	6.1698	5.0461	4.9041	13.4868	5.3652	42.668
PMR-48	YNWS202004	2.3333	1.3810	1.5781	1.4537	5.0486	2.3144	14.1091
PMR-49	YNWS202005	14.0761	13.6632	12.5066	13.3863	54.2692	22.3430	130.2444
PMR-50	YNQJ202001	5.0686	3.6480	3.3502	3.2861	7.9063	3.5563	26.8155
PMR-51	YNQJ202002	10.7646	10.9368	8.2626	8.9494	23.5746	9.7494	72.2374
PMR-52	YNQJ202003	15.1922	14.2283	10.7753	10.8187	22.6107	9.4178	83.043
PMR-53	YNQJ202004	6.7141	5.1772	4.8731	4.7347	9.3661	4.3819	35.2471
PMR-54	YNQJ202005	0.7319	0.4085	0.9288	0.8719	3.8983	1.5935	8.4329
PMR-55	YNQJ20201101	26.9930	18.3799	25.2812	24.7760	86.9190	32.0650	214.4141
PMR-56	GDDQ202109	4.0790	1.6728	2.8613	2.6000	3.6367	1.8888	16.7386
PMR-57	GDDQ2020	39.8238	38.4998	50.6853	44.1359	113.3277	73.5961	360.0686
PMR-58	GDDQ-1NQ-1	8.4463	10.7348	8.2408	9.8430	38.2572	13.6106	89.1327
PMR-59	GDDQ-1NQ-2	4.1493	4.8934	4.2004	4.7226	16.0713	6.3531	40.3901
PMR-60	GDDQ-1NQ-3	4.9853	5.7515	4.3289	4.8566	14.4549	5.1687	39.5459
PMR-61	GDDQ-1NQ-4	3.1272	3.1105	2.8775	3.1513	10.3475	3.6803	26.2943
PMR-62	GDDQ-1NQ-5	7.7492	9.9420	8.5174	9.8373	44.4808	16.2467	96.7734
PMR-63	GDDQ-2NQ-1	5.0700	6.3349	5.7655	6.6218	29.3260	10.9279	64.0461
PMR-64	GDDQ-2NQ-2	4.2366	4.5830	4.1882	4.8453	16.9752	7.0166	41.8449
PMR-65	GDDQ-2NQ-3	6.5193	8.1737	6.4709	7.4134	28.9470	11.2509	68.7752
PMR-66	GDDQ-2NQ-4	3.6876	2.4314	2.6999	3.1484	16.5265	6.0976	34.5914
PMR-67	GDDQ-2NQ-5	4.7314	5.6643	4.5871	5.1391	15.9686	5.7854	41.8759
PMR-68	GDDQ-3NQ-1	2.6865	3.0152	3.2494	3.7024	16.0974	5.5573	34.3082
PMR-69	GDDQ-3NQ-2	2.2473	2.4765	2.7302	3.1680	14.6158	5.1880	30.4258
PMR-70	GDDQ-3NQ-3	2.9730	3.8137	3.8154	4.2450	19.5624	6.8140	41.2235
PMR-71	GDDQ-3NQ-4	2.1730	2.4436	3.0138	3.3788	16.9050	5.7368	33.651
PMR-72	GDDQ-3NQ-5	1.0927	1.1420	1.4584	1.5930	7.9614	2.8537	16.1012
PMR-73	GDDQ-4NQ-1	1.1056	1.2292	1.3755	1.6341	7.7680	2.8814	15.9938
PMR-74	GDDQ-4NQ-2	2.8829	3.7151	3.5472	4.2084	17.9742	6.1962	38.524
PMR-75	GDDQ-4NQ-3	3.0047	3.5324	3.1769	3.6740	14.3967	4.9574	32.7421
PMR-76	GDDQ-4NQ-4	2.4191	2.6001	2.6129	2.9144	11.4294	3.4791	25.455
PMR-77	GDDQ-4NQ-5	2.7111	3.3084	3.3873	3.7605	16.3031	5.6654	35.1358
PMR-78	GDDQ-5NQ-1	2.9051	3.6573	3.3128	3.8702	15.6144	6.2419	35.6017
PMR-79	GDDQ-5NQ-2	2.2383	2.6039	2.5724	2.9636	11.9649	4.6200	26.9631
PMR-80	GDDQ-5NQ-3	6.9379	0.8984	7.6715	8.9943	34.5678	13.2128	72.2827
PMR-81	GDDQ-5NQ-4	1.8152	2.2026	2.2054	2.4565	10.0729	3.8902	22.6428

PMR-82	GDDQ-5NQ-5	2.1998	2.2092	2.1214	2.3251	8.2151	3.3458	20.4164
PMR-83	GDDQ-6NQ-1	1.5630	1.6442	1.9100	2.1454	11.3631	4.4167	23.0424
PMR-84	GDDQ-6NQ-2	1.4284	1.7851	2.2789	2.5179	13.1420	5.2645	26.4168
PMR-85	GDDQ-6NQ-3	1.2656	1.1414	1.3907	1.5105	7.0037	2.6303	14.9422
PMR-86	GDDQ-6NQ-4	1.4171	1.4120	1.3003	1.4128	5.7009	2.1451	13.3882
PMR-87	GDDQ-6NQ-5	0.8313	0.6967	0.7947	0.8454	3.8302	1.5249	8.5232
PMR-88	GDDQ-5NC-1	1.2186	1.3126	1.6989	1.9310	9.1994	3.6842	19.0447
PMR-89	GDDQ-5NC-2	3.8728	4.1589	4.0331	4.5712	14.3139	5.9859	36.9358
PMR-90	GDDQ-5NC-3	2.1411	1.8629	1.9171	2.1268	6.4340	2.6741	17.156
PMR-91	GDDQ-5NC-4	0.6700	0.5645	0.8043	0.8872	4.2172	1.4918	8.635
PMR-92	GDDQ-5NC-5	3.3708	3.9064	3.7186	4.2019	15.0069	6.0827	36.2873
PMR-93	GDDQ-5NX-1	0.1153	0.0189	0.1033	0.0631	0.1990	0.1017	0.6013
PMR-94	GDDQ-5NX-2	2.6308	2.3429	2.4170	2.4832	5.7160	2.2472	17.8371
PMR-95	GDDQ-5NX-3	0.2385	0.1278	0.1844	0.1617	0.4144	0.1777	1.3045
PMR-96	GDDQ-5NX-4	0.8055	0.7023	0.6889	0.6730	1.7798	0.6967	5.3462
PMR-97	GDDQ-5NX-5	3.2798	3.4518	2.9309	3.3186	8.9035	3.6217	25.5063

[a] The sum of the contents of the six compounds.

Table S2. Contents of 6 compounds in PMR and PMRP collected from herb markets and pharmaceutical companies.

Samples	Batch Number		Contents of analytes (μg/g)						
			**1**	**2**	**3**	**4**	**5**	**6**	**total**[△]
PRMP-01	AHBZSC202001Z	a	0.2924	0.3140	0.4902	0.4709	0.9598	0.6729	3.2002
		b	1.6951	1.5458	1.1980	1.1230	1.5671	1.1757	8.3047
PRMP-02	AHBZSC202002Z	a	0.6855	0.7613	1.1347	1.2025	2.7927	2.0127	8.5894
		b	1.9509	1.6996	1.6652	1.6107	2.7997	2.1923	11.9184
PRMP-03	AHBZSC202003Z	a	0.3441	0.3901	0.6264	0.6697	1.4463	1.0651	4.5417
		b	0.8297	0.7630	0.7882	0.7378	1.3794	1.0677	5.5658
PRMP-04	AHBZSC202004Z	a	0.3413	0.4162	0.5560	0.5921	1.0822	0.7824	3.7702
		b	1.1471	1.0549	0.8601	0.7798	1.0346	0.6899	5.5664
PRMP-05	AHBZSC202005Z	a	0.0276	0.0920	0.1637	0.1733	0.4604	0.3664	1.2834
		b	0.3268	0.3168	0.2428	0.2297	0.3703	0.3836	1.87
PRMP-06	SW01	a	0.0090	0.0078	0.1406	0.2749	0.5127	0.2421	1.1871
		b	0.0201	0.0070	0.1583	0.3452	0.2890	0.0110	0.8306
PRMP-07	SW02	a	0.0072	0.0058	0.0838	0.1300	0.1939	0.0035	0.4242
		b	0.0132	0.0039	0.0460	0.0991	0.0637	0.0042	0.2301
PRMP-08	SW03	a	0.0399	0.0124	0.1844	0.3329	0.5204	0.2329	1.3229
		b	0.0192	0.0055	0.1528	0.3045	0.3112	0.0104	0.8036
PRMP-09	SW04	a	0.0144	0.0273	0.1253	0.2111	0.2925	0.0610	0.7316
		b	0.0176	0.0045	0.0976	0.0793	0.1434	0.0065	0.3489
PRMP-10	SW05	a	0.0076	0.0223	0.0942	0.1569	0.2396	0.0047	0.5253
		b	0.0133	0.0034	0.0486	0.0991	0.0741	0.0049	0.2434
PRMP-11	SW06	a	0.0771	0.4266	0.2059	0.3647	0.4167	0.1503	1.6413
		b	0.0341	0.0066	0.1634	0.3234	0.2207	0.0081	0.7563
PRMP-12	GXYLSC202001Z	a	ND *	ND *	ND *	ND *	ND *	ND *	0
		b	ND *	ND *	ND *	ND *	ND *	ND *	0
PRMP-13	SXXASC202001Z	a	0.6795	0.6708	1.1730	2.2552	2.8511	1.8511	9.4807
		b	10.6146	7.8054	5.8071	8.2531	5.3551	2.7923	40.6276
PRMP-14	HBAGSC202001Z	a	2.3755	2.3992	2.9439	6.0064	5.2643	3.9680	22.9573
		b	2.8126	2.9609	2.9360	6.0261	4.4363	3.0131	22.185
PRMP-15	GDDQ	a	0.3665	0.4871	0.3177	0.7658	0.3429	0.1918	2.4718
		b	17.5481	22.6380	14.3351	37.3134	17.0336	16.0577	124.9259
PRMP-16	AHWST01	a	0.0265	0.0331	0.0743	0.0617	0.0662	0.0759	0.3377

		b	0.6928	0.6422	0.3911	0.3604	0.1764	0.1373	2.4002
PRMP-17	AHWST02	a	0.0423	0.0462	0.1050	0.1022	0.1637	0.1241	0.5835
		b	0.7246	0.7303	0.4463	0.4551	0.3368	0.2848	2.9779
PRMP-18	AHWST03	a	0.3389	0.3375	0.4372	0.4587	0.7894	0.5599	2.9216
		b	4.0049	3.9154	2.1743	2.2890	1.5454	1.1733	15.1023
PRMP-19	AHWST04	a	0.2253	0.2290	0.2897	0.3028	0.5039	0.3700	1.9207
		b	3.4735	3.1429	1.7723	1.8280	1.3079	0.9631	12.4877
PRMP-20	AHWST05	a	0.1864	0.1811	0.2507	0.2772	0.4054	0.3242	1.625
		b	2.8894	2.5030	1.4656	1.3591	0.9173	0.5630	9.6974
PRMP-21	AHWST06	a	0.1225	0.1236	0.2084	0.2068	0.3719	0.2445	1.2777
		b	2.0302	1.8442	1.0533	1.0160	0.6315	0.5426	7.1178
PRMP-22	AHWST07	a	0.1896	0.1601	0.3314	0.2949	0.5890	0.3570	1.922
		b	0.5931	0.5730	0.4903	0.4693	0.5081	0.3915	3.0253
PRMP-23	2015G0624	a	0.8986	1.5427	1.4309	1.5417	1.8641	1.4107	8.6887
		b	7.9831	9.6812	4.1572	5.4302	3.2718	2.2810	32.8045
PRMP-24	2015G0057	a	6.0446	5.0787	8.1804	8.5416	11.9543	8.2166	48.0162
		b	19.2698	22.0699	16.0484	19.4401	17.0633	13.4773	107.3688
PRMP-25	2015G0371	a	5.9810	5.0892	8.9669	9.4526	13.7182	8.9840	52.1919
		b	13.5853	14.1080	13.1322	14.3224	14.4622	11.2716	80.8817
PRMP-26	2015G0251	a	13.7428	15.5444	9.9900	12.2314	9.3771	7.1689	68.0546
		b	64.7599	67.0548	43.4503	53.3514	37.9111	30.6754	297.2029
PRMP-27	2015G0162	a	9.3711	10.3503	5.2289	5.9066	4.2295	3.4136	38.5
		b	8.7480	9.9210	4.8213	5.2348	2.9415	1.8531	33.5197
PRMP-28	2015G0239	a	8.5469	10.7631	5.4469	7.1152	5.1532	3.8169	40.8422
		b	47.8774	53.3681	34.8343	46.0596	33.8902	32.1337	248.1633
PRMP-29	2015G0375	a	8.6649	9.3339	9.2268	10.3908	13.8583	9.9376	61.4123
		b	13.8750	15.6238	13.2851	15.0111	16.6278	14.0312	88.454
PRMP-30	2015G0625	a	8.3069	8.3127	16.8973	18.1693	48.4669	35.0726	135.2257
		b	23.0288	19.0677	24.1761	22.5738	48.8397	35.1981	172.8842
PRM-98	20211122-GSJN-S01	a	9.5383	6.8713	4.4099	4.4243	3.3176	2.5253	31.0867
		b	21.7565	21.0313	11.0361	13.8539	26.2990	8.7785	102.7553
PRM-99	20211122-GSJN-S02	a	8.9198	6.2178	4.2431	4.0237	3.0219	2.3070	28.7333
		b	20.4897	20.3125	10.7350	13.2151	26.2901	7.9001	98.9425
PRMP-31	20211122-GSJN-Z01	a	0.4231	0.3768	0.5226	0.5147	0.9879	0.6963	3.5214
		b	2.9650	3.6230	1.8921	2.3509	2.1666	1.4921	14.4897
PRMP-32	20211122-GSJN-Z02	a	0.4729	0.3675	0.6770	0.6486	1.0477	0.6756	3.8893
		b	2.9066	3.3712	1.5753	2.4692	1.8915	1.2646	13.4784
PRMP-33	20211122-GSJN-Z03	a	0.2649	0.2397	0.4164	0.3744	0.9039	0.6542	2.8535
		b	1.1005	1.0646	0.7893	0.7610	1.3441	0.8788	5.9383
PRMP-34	20211122-GSJN-Z04	a	0.1476	0.1292	0.2534	0.2079	0.5603	0.3102	1.6086
		b	0.9127	0.8188	0.6159	0.5200	0.7842	0.5030	4.1546
PMP-100	210901-1	a	110.8115	89.2393	60.7679	65.5450	65.2657	51.1096	442.739
		b	35.1888	33.4687	13.6371	21.0371	16.6803	14.0145	134.0265
PMP-101	210901-2	a	109.5964	81.2675	64.2949	68.0623	66.2652	50.8741	440.3604
		b	28.4709	26.7475	15.5180	18.4579	15.1173	12.2221	116.5337
PRMP-35	210901-ZHSW-01	a	0.7041	0.5758	0.9470	0.9187	1.1473	0.7470	5.0399
		b	9.7064	12.6004	5.7666	8.6589	4.0118	3.1831	43.9272
PRMP-36	210901-ZHSW-02	a	1.1790	0.9241	1.5069	1.5232	1.8021	1.1923	8.1276
		b	10.2154	13.2912	6.3208	9.3557	4.3791	4.1155	47.6777
PRMP-37	210901-ZHSW-03	a	0.4006	0.4337	0.6855	0.6778	1.2504	0.7832	4.2312
		b	1.7872	1.4211	1.1617	0.9790	2.8423	0.9344	9.1257
PRMP-38	210901-ZHSW-04	a	0.3237	0.2848	0.5146	0.4821	0.8723	0.5388	3.0163
		b	1.8007	1.3736	1.0419	0.8247	2.7158	0.6860	8.4427

PMP-102	20211125-GSLM-S01	a	17.0679	14.5050	9.0876	6.2936	5.3782	4.0498	56.3821
		b	38.6649	40.5193	18.7000	21.8633	9.3863	9.6624	138.7962
PRMP-39	20211125-GSLM-Z01	a	0.5539	0.4935	0.7721	0.7279	1.1970	0.7283	4.4727
		b	8.2831	8.9139	3.6980	3.7416	2.3123	4.2025	31.1514
PRMP-40	20211125-GSLM-Z02	a	0.0105	0.0628	0.0842	0.0745	0.2354	0.1844	0.6518
		b	2.6300	2.1580	0.9343	0.6931	0.4627	0.2889	7.167
PMP-103	20211125-NXMD-S01	a	3.9726	4.2099	1.7534	1.9107	0.4587	0.9961	13.3014
		b	10.1445	12.0110	5.0818	6.1240	1.6138	2.8649	37.84
PMP-104	20211125-NXMD-S02	a	3.4603	3.7047	1.6167	1.7320	0.3367	0.9656	11.816
		b	9.3674	10.9149	4.7115	5.7122	1.5368	2.6325	34.8753
PRMP-41	20211125-NXMD-Z01	a	0.6221	0.6292	0.8714	0.8857	1.6200	0.9862	5.6146
		b	9.0394	11.4930	5.7548	6.9258	5.0817	3.6129	41.9076
PRMP-42	20211125-NXMD-Z02	a	0.5620	0.5758	0.7386	0.7648	1.1881	0.8109	4.6402
		b	3.8372	4.8570	2.2989	2.8650	2.1100	1.3989	17.367
PRMP-43	20211125-NXMD-Z03	a	0.7753	0.6423	0.9090	0.9119	1.3366	0.8581	5.4332
		b	3.6593	4.5258	2.2215	2.7276	2.0168	1.4068	16.5578
PRMP-44	20211125-NXMD-Z04	a	0.2921	0.3133	0.4455	0.4599	0.7954	0.5617	2.8679
		b	2.1871	2.1818	0.8462	1.0633	0.9216	0.6015	7.8015
PRMP-45	20211125-NXMD-Z05	a	0.2982	0.3191	0.4083	0.4071	0.6881	0.4563	2.5771
		b	1.6812	1.6108	0.7975	0.8072	0.6677	0.4684	6.0328
PRMP-46	20211125-NXMD-Z06	a	0.1925	0.2170	0.2865	0.2893	0.4430	0.3417	1.77
		b	1.4667	1.5280	0.6868	0.6324	0.4599	0.3526	5.1264
PRMP-47	20211125-NXMD-Z07	a	0.1096	0.1438	0.2170	0.2015	0.3580	0.2787	1.3086
		b	2.1824	2.1786	0.9737	0.8895	0.5760	0.4152	7.2154
PRMP-48	20211125-NXMD-Z08	a	0.3844	0.3813	0.4682	0.6027	1.0889	0.6636	3.5891
		b	2.0504	2.0275	1.2112	1.1527	1.2055	0.8873	8.5346
PRMP-49	20211125-NXMD-Z09	a	0.2739	0.2703	0.4321	0.2925	0.7841	0.5118	2.5647
		b	1.9475	1.9160	1.0435	0.9482	0.7898	0.5333	7.1783
PMP-105	20211129-JXJSTQT-S01	a	8.0383	6.6111	4.5575	4.4354	3.2835	2.6294	29.5552
		b	16.8478	17.8481	10.0183	12.3515	7.7830	7.1118	71.9605
PMP-106	20211129-JXJSTQT-S02	a	7.3113	5.9471	4.1045	4.1419	3.0161	2.4577	26.9786
		b	14.6390	15.1368	8.9976	10.8256	7.6078	6.1482	63.355
PMP-107	20211129-JXJSTQT-S03	a	6.3124	5.2137	0.3663	3.6448	2.5658	2.0656	20.1686
		b	13.3661	14.5035	8.1648	9.7667	6.7224	0.5479	53.0714
PRMP-50	20211129-JXJSTQT-Z01	a	1.6930	1.4737	1.4912	1.5759	1.7862	1.2089	9.2289
		b	5.0023	6.1052	3.3191	4.7242	3.3439	2.8591	25.3538
PRMP-51	20211129-JXJSTQT-Z02	a	0.8615	0.7525	0.8756	0.8972	1.2490	0.7921	5.4279
		b	3.3634	4.3430	2.1705	3.1590	2.3862	6.4389	21.861
PRMP-52	20211129-JXJSTQT-Z03	a	0.7735	0.7292	0.8476	0.8719	1.2648	0.8535	5.3405
		b	2.7739	3.7426	1.9561	2.7397	2.2331	1.8595	15.3049
PRMP-53	20211129-JXJSTQT-Z04	a	0.4397	0.4462	0.6415	0.6589	1.0305	0.7154	3.9322
		b	0.9125	0.9086	0.8276	0.8387	1.1262	0.8165	5.4301
PRMP-54	20211129-JXJSTQT-Z05	a	0.4953	0.5069	0.7290	0.7469	1.1707	0.8400	4.4888
		b	0.4953	0.5069	0.7290	0.7469	1.1707	0.8400	4.4888
PRMP-55	20211129-JXJSTQT-	a	0.5180	0.5301	0.6364	0.7886	1.2573	0.8430	4.5734

	Z06								
		b	0.5180	0.5301	0.6364	0.7886	1.2573	0.8430	4.5734
PMP-108	20211203-AGYPS01	a	23.4415	23.6801	17.4026	19.2068	23.2315	16.3325	123.295
		b	66.3944	71.4089	42.1844	47.3991	41.2125	28.6417	297.241
PRMP-56	20211203-AGYPZ01	a	0.2132	0.2390	0.2325	0.2383	0.3264	0.2126	1.462
		b	0.6126	0.8095	0.5068	0.6396	0.5631	0.4442	3.5758
PMP-109	20211203-BZLDS01	a	12.8844	13.3425	6.8638	7.3696	6.8254	5.1375	52.4232
		b	34.1335	34.5918	17.3008	19.2733	28.7589	10.5963	144.6546
PMP-110	20211203-BZLDS02	a	5.1231	5.0238	2.4361	2.9265	3.2963	2.2619	21.0677
		b	16.6959	17.5800	9.0236	10.2709	25.7318	5.9877	85.2899
PRMP-57	20211203-BZLDZ01	a	0.3301	0.2682	0.4310	0.3952	0.8133	0.4847	2.7225
		b	0.7294	0.7068	0.6026	0.5624	2.6628	0.6192	5.8832
PRMP-58	20211203-BZLDZ02	a	0.1434	0.1268	0.2266	0.1930	0.4440	0.2264	1.3602
		b	0.4120	0.4536	0.3406	0.2610	2.4639	0.3116	4.2427
PMP-111	20211222-AHWSS01	a	9.9610	8.3938	7.9430	8.3028	8.2522	6.2447	49.0975
		b	19.5809	21.1166	13.2408	16.7674	11.4959	10.2873	92.4889
PRMP-59	20211222-AHWSZ01	a	0.5812	0.4994	0.6757	0.7360	1.2311	0.7252	4.4486
		b	4.8122	7.3807	3.0067	4.7787	2.3619	1.9843	24.3245
PRMP-60	20211222-AHWSZ02	a	1.5772	1.1321	2.5456	2.4493	3.0335	2.0031	12.7408
		b	11.9119	16.2899	7.6002	10.6428	5.9005	5.2866	57.6319
PRMP-61	20211222-AHWSZ03	a	0.7742	0.5759	0.9751	0.9004	1.3555	0.9340	5.5151
		b	5.6427	7.8517	3.7808	5.2107	3.0722	2.8130	28.3711
PRMP-62	20211222-AHWSZ04	a	0.4397	0.3820	0.5095	0.5786	0.9807	0.6468	3.5373
		b	5.5663	8.0215	3.2687	3.8327	2.4904	2.3194	25.499
PRMP-63	20211222-AHWSZ05	a	0.5621	0.4886	0.6596	0.6028	0.8832	0.5974	3.7937
		b	5.5663	8.0215	3.2687	3.8327	2.4904	2.3194	25.499
PRMP-64	20211222-AHWSZ06	a	2.4813	2.2017	2.6840	2.7579	3.3775	2.3193	15.8217
		b	5.8923	7.7525	4.8339	6.2915	3.9179	3.5849	32.273
PMP-112	20220104-ZJTJTS01	a	7.9084	5.7814	3.9883	4.0798	7.5635	2.7959	32.1173
		b	168.2470	183.6098	96.7821	115.6580	171.1238	83.1678	818.5885
PMP-113	20220104-ZJTJTS02	a	4.5252	2.9416	2.0081	2.0314	3.7681	1.5587	16.8331
		b	105.7654	112.0035	59.8033	73.1122	101.1063	47.9756	499.7663
PRMP-65	20220104-ZJTJTZ01	a	0.1803	0.1191	0.3239	0.2680	1.0985	0.4383	2.4281
		b	1.6618	1.2827	1.3453	1.1593	3.3503	1.2259	10.0253
PRMP-66	20220104-ZJTJTZ02	a	0.2378	0.1913	0.3835	0.3397	1.4061	0.4890	3.0474
		b	2.4441	2.1331	1.8033	1.6068	4.6884	1.7011	14.3768
PRMP-67	20220104-ZJTJTZ03	a	0.1986	0.1455	0.3437	0.2908	1.3083	0.4630	2.7499
		b	1.8810	1.5830	1.4473	1.2556	3.8051	1.4283	11.4003
PRMP-68	20220104-ZJTJTZ04	a	0.1674	0.1138	0.3001	0.2508	0.9819	0.4153	2.2293
		b	1.5234	1.3277	1.1106	1.0160	2.8993	1.1180	8.995
PRMP-69	20220104-ZJTJTZ05	a	0.0988	0.0657	0.2036	0.1724	0.7400	0.2866	1.5671
		b	1.3805	1.2493	0.8823	0.7537	2.4458	0.8661	7.5777
PRMP-70	20220104-ZJTJTZ06	a	0.1155	0.0886	0.2259	0.1943	0.8371	0.3399	1.8013
		b	1.5746	1.5113	0.8547	0.7667	2.2989	0.9470	7.9532
PRMP-71	20220104-ZJTJTZ07	a	0.1161	0.0756	0.2126	0.1766	0.7220	0.2844	1.5873
		b	0.5244	0.3083	0.5403	0.4493	1.6622	0.5864	4.0709
PRMP-72	20220104-ZJTJTZ08	a	0.1781	0.1253	0.2899	0.2493	1.0819	0.3827	2.3072
		b	1.9596	1.7714	0.8134	0.7190	2.0498	0.8016	8.1148
PRMP-73	20220104-ZJTJTZ09	a	0.3224	0.2509	0.4723	0.4395	1.4122	0.5618	3.4591
		b	1.0741	0.7340	1.1487	0.9822	3.1347	1.1161	8.1898
PRMP-74	20220104-ZJTJTZ10	a	0.1300	0.0806	0.2281	0.1802	0.7311	0.2765	1.6265
		b	0.6259	0.3639	0.6156	0.5199	1.6216	0.5995	4.3464
PRMP-75	20220104-ZJTJTZ11	a	0.3139	0.2261	0.5019	0.4631	1.8685	0.6598	4.0333

		b	1.0700	0.8014	1.1843	1.0001	2.9821	1.2364	8.2743
PRMP-76	20220104-ZJTJTZ12	a	0.1169	0.0557	0.2038	0.1538	0.7024	0.2367	1.4693
		b	0.5400	0.2707	0.5324	0.3858	1.6724	0.5744	3.9757

[a] Content of six compounds before hydrolysis. [b] Content of six compounds after hydrolysis. △ The sum of the contents of the six compounds. * ND, under limits of quantitation.

Table S3. Contents of 6 compounds in different processed PMRP.

**Samples**		**Contents of analytes (μg/g)**						
		**1**	**2**	**3**	**4**	**5**	**6**	**Total**△
PMR-12	a	64.0366	61.9686	63.8661	67.3944	46.2105	31.5802	335.0564
	b	120.4388	135.7185	107.3601	123.1741	100.2338	75.8933	662.8186
PMRP-Q_{2h}	a	18.9556	16.3818	30.4316	31.8016	48.8257	32.6669	179.0632
	b	70.2607	102.1134	63.7564	90.7059	87.7598	70.8167	485.4129
PMRP-Q_{4h}	a	13.0024	15.2831	24.3831	26.8371	51.1396	32.3053	162.9506
	b	48.5066	57.2735	40.0598	5.4654	52.5756	41.2789	245.1598
PMRP-Q_{8h}	a	6.2447	6.3921	14.3036	15.7129	34.0226	21.1398	97.8157
	b	20.7371	27.4309	25.0324	29.8011	43.8235	28.8546	175.6796
PMRP-Q_{12h}	a	5.3355	5.0120	11.1662	12.1042	23.5104	14.5950	71.7233
	b	16.1662	17.2213	17.4848	18.2370	27.1005	15.6690	111.8788
PMRP-Q_{18h}	a	3.7783	3.1680	6.7140	7.1954	14.1389	8.6923	43.6869
	b	8.7287	7.1682	8.2284	7.6362	13.1687	7.2577	52.1879
PMRP-Q_{24h}	a	3.0092	2.8972	6.7266	7.2435	19.9856	11.8687	51.7308
	b	5.6497	3.6530	6.6779	6.1935	17.1603	9.9430	49.2774
PMRP-Q_{30h}	a	1.7665	1.3834	3.4854	3.7352	10.2495	6.3358	26.9558
	b	3.4439	1.6435	3.1820	2.7095	8.7468	4.9429	24.6686
PMRP-Q_{36h}	a	0.7297	0.3176	1.2856	1.3326	4.2633	2.6219	10.5507
	b	1.8461	0.2523	0.7231	0.4232	3.0572	1.9083	8.2102
PMRP-Q_{48h}	a	0.3402	0.0280	0.2321	0.1881	1.9912	1.3101	4.0897
	b	0.5001	0.0314	0.0491	0.0657	1.6723	0.5157	2.8343
PMRP-H_{2h}	a	20.9902	21.8696	28.4251	31.9708	39.6297	28.1394	171.0248
	b	63.4527	68.8679	49.1361	73.4823	62.4058	51.7436	369.0884
PMRP-H_{4h}	a	8.1951	10.1411	18.9248	21.1948	30.7772	21.0535	110.2865
	b	65.6631	84.5382	53.3097	77.1076	65.1215	51.8454	397.5855
PMRP-H_{8h}	a	12.1234	10.6589	17.9266	20.6275	38.1601	23.0745	122.571
	b	37.5893	39.2741	32.1817	40.0811	54.7227	33.7200	237.5689
PMRP-H_{12h}	a	6.4994	5.7148	11.5422	12.9585	28.9288	17.6924	83.3361
	b	16.1651	14.1932	16.0455	17.0635	29.7911	31.5844	124.8428
PMRP-H_{18h}	a	5.2238	4.5126	9.1178	10.2694	24.1647	14.7018	67.9901
	b	11.5213	8.4062	11.4125	11.0249	22.1878	36.7544	101.3071
PMRP-H_{24h}	a	2.2879	1.8355	4.0756	4.5144	12.6265	6.0676	31.4075
	b	5.5322	3.1120	4.3918	3.8561	7.4316	15.1691	39.4928
PMRP-H_{30h}	a	0.3050	0.6112	1.4130	1.5472	6.5773	3.1770	13.6307
	b	2.4251	1.0572	1.8457	0.9974	4.4050	7.9425	18.6729
PMRP-H_{36h}	a	0.9028	1.0557	2.2873	2.4509	5.4007	4.2033	16.3007
	b	1.2280	0.9057	1.8306	1.5343	5.6702	10.5082	21.677
PMRP-H_{48h}	a	0.0237	0.0246	0.1125	0.0615	3.9789	1.1233	5.3245
	b	0.5041	0.0397	0.0732	0.0714	0.2355	2.8083	3.7322
PMR-57	a	39.8238	38.4998	50.6853	44.1359	113.3277	73.5961	360.0686
	b	194.2306	215.0783	157.6543	194.0987	231.6045	156.7588	1149.4252
PMRP-$JZJZ_1$	a	0.0076	0.0137	0.0062	0.0089	0.0140	0.0071	0.0575
	b	20.8615	25.2909	15.1541	20.9782	17.2611	12.1074	111.6532
PMRP-$JZJZ_2$	a	ND *	0.0121	ND *	0.0043	0.0061	0.0050	0.0275
	b	11.4996	10.7753	6.3152	5.8715	4.8142	1.7191	40.9949

PMRP-JZJZ3	a	ND *	0.0059	ND *	ND *	0.0084	0.0054	0.0197
	b	6.3052	3.1961	2.1552	0.9323	1.0926	0.2430	13.9244
PMRP-JZJZ4	a	ND *	ND *	ND *	ND *	ND *	ND *	0
	b	0.5496	0.1522	0.0323	0.0140	0.0283	0.0076	0.784
PMRP-JZJZ5	a	ND *	ND *	ND *	ND *	ND *	ND *	0
	b	0.0361	0.0081	ND *	ND *	ND *	ND *	0.0442
PMRP-JZJZ6	a	ND *	ND *	ND *	ND *	0.0068	0.0035	0.0103
	b	ND *	ND *	ND *	ND *	0.0171	0.0080	0.0251
PMRP-JZJZ7	a	ND *	ND *	ND *	ND *	ND *	ND *	0
	b	0.0149	0.0100	0.0109	0.0145	0.0221	0.0126	0.085
PMRP-JZJZ8	a	ND *	ND *	ND *	ND *	ND *	ND *	0
	b	0.0131	0.0105	0.0129	0.0169	0.0243	0.0186	0.0963
PMRP-JZJZ9	a	ND *	ND *	ND *	ND *	ND *	ND *	0
	b	0.0344	0.1883	0.0352	0.0501	0.0678	0.1770	0.5528
PRMP-DZ	a	0.1385	0.3255	0.5710	0.7077	1.9649	1.7291	5.4367
	b	13.4136	16.0378	10.4085	14.3622	13.8245	9.5942	77.6408

[a] Content of six compounds before hydrolysis. [b] Content of six compounds after hydrolysis. △ The sum of the contents of the six compounds. * ND, under limits of quantitation.

Table S4. Sample collection information of PMR.

Samples	Batch Number	Source	Collect Time
PMR-01	AHBZ202001	Bozhou, Anhui Province, China	2020.8.7
PMR-02	AHBZ202002	Bozhou, Anhui Province, China	2020.8.7
PMR-03	AHBZ202003	Bozhou, Anhui Province, China	2020.8.7
PMR-04	AHBZ202004	Bozhou, Anhui Province, China	2020.8.7
PMR-05	AHBZ202005	Bozhou, Anhui Province, China	2020.8.7
PMR-06	AHFY202001	Bozhou, Anhui Province, China	2020.8.7
PMR-07	AHFY202002	Bozhou, Anhui Province, China	2020.8.7
PMR-08	AHFY202003	Bozhou, Anhui Province, China	2020.8.7
PMR-09	AHFY202004	Bozhou, Anhui Province, China	2020.8.7
PMR-10	AHFY202005	Bozhou, Anhui Province, China	2020.8.7
PMR-11	GDGZ202002	Gaozhou, Guangdong Province, China	2020.8.3
PMR-12	GDGZ202003	Gaozhou, Guangdong Province, China	2020.8.3
PMR-13	GDGZ202004	Gaozhou, Guangdong Province, China	2020.8.3
PMR-14	GDGZ202005	Gaozhou, Guangdong Province, China	2020.8.3
PMR-15	SCDZ202001	Dazhou, Sichuan Province, China	2020.12.29
PMR-16	SCDZ202002	Dazhou, Sichuan Province, China	2020.12.29
PMR-17	SCDZ202003	Dazhou, Sichuan Province, China	2020.12.29
PMR-18	SCDZ202004	Dazhou, Sichuan Province, China	2020.12.29
PMR-19	SCDZ202005	Dazhou, Sichuan Province, China	2020.12.29
PMR-20	SCYB202001	Yibin, Sichuan Province, China	2020.8.20
PMR-21	SCYB202002	Yibin, Sichuan Province, China	2020.8.20
PMR-22	SCYB202003	Yibin, Sichuan Province, China	2020.8.20
PMR-23	SCYB202004	Yibin, Sichuan Province, China	2020.8.20
PMR-24	SCYB202005	Yibin, Sichuan Province, China	2020.8.20
PMR-25	GZKL202001	Kaili, Guizhou Province, China	2020.11.17
PMR-26	GZKL202002	Kaili, Guizhou Province, China	2020.11.17
PMR-27	GZKL202003	Kaili, Guizhou Province, China	2020.11.17
PMR-28	GZKL202004	Kaili, Guizhou Province, China	2020.11.17
PMR-29	GZKL202005	Kaili, Guizhou Province, China	2020.11.17

PMR-30	GZBJ202001	Bijie, Guizhou Province, China	2020.11.17
PMR-31	GZBJ202002	Bijie, Guizhou Province, China	2020.11.17
PMR-32	GZBJ202003	Bijie, Guizhou Province, China	2020.11.17
PMR-33	GZBJ202004	Bijie, Guizhou Province, China	2020.11.17
PMR-34	GZBJ202005	Bijie, Guizhou Province, China	2020.11.17
PMR-35	GZAS202001	Anshun, Guizhou Province, China	2020.11.17
PMR-36	GZAS202002	Anshun, Guizhou Province, China	2020.11.17
PMR-37	GZAS202003	Anshun, Guizhou Province, China	2020.11.17
PMR-38	GZAS202004	Anshun, Guizhou Province, China	2020.11.17
PMR-39	GZAS202005	Anshun, Guizhou Province, China	2020.11.17
PMR-40	GZTR202001	Tongren, Guizhou Province, China	2020.11.17
PMR-41	GZTR202002	Tongren, Guizhou Province, China	2020.11.17
PMR-42	GZTR202003	Tongren, Guizhou Province, China	2020.11.17
PMR-43	GZTR202004	Tongren, Guizhou Province, China	2020.11.17
PMR-44	GZTR202005	Tongren, Guizhou Province, China	2020.11.17
PMR-45	YNHH20201101	Honghe, Yunnan Province, China	2020.11.13
PMR-46	YNWS202002	Wenshan, Yunnan Province, China	2020.8.12
PMR-47	YNWS202003	Wenshan, Yunnan Province, China	2020.8.12
PMR-48	YNWS202004	Wenshan, Yunnan Province, China	2020.8.12
PMR-49	YNWS202005	Wenshan, Yunnan Province, China	2020.8.12
PMR-50	YNQJ202001	Qujing, Yunnan Province, China	2020.8.12
PMR-51	YNQJ202002	Qujing, Yunnan Province, China	2020.8.12
PMR-52	YNQJ202003	Qujing, Yunnan Province, China	2020.8.12
PMR-53	YNQJ202004	Qujing, Yunnan Province, China	2020.8.12
PMR-54	YNQJ202005	Qujing, Yunnan Province, China	2020.8.12
PMR-55	YNQJ20201101	Qujing, Yunnan Province, China	2020.11.13
PMR-56	GDDQ202109	DeQing, Guangdong Province, China	Unknown
PMR-57	GDDQ2020	DeQing, Guangdong Province, China	Unknown
PMR-58	GDDQ-1NQ-1	DeQing, Guangdong Province, China	2021.11.29
PMR-59	GDDQ-1NQ-2	DeQing, Guangdong Province, China	2021.11.29
PMR-60	GDDQ-1NQ-3	DeQing, Guangdong Province, China	2021.11.29
PMR-61	GDDQ-1NQ-4	DeQing, Guangdong Province, China	2021.11.29
PMR-62	GDDQ-1NQ-5	DeQing, Guangdong Province, China	2021.11.29
PMR-63	GDDQ-2NQ-1	DeQing, Guangdong Province, China	2021.11.29
PMR-64	GDDQ-2NQ-2	DeQing, Guangdong Province, China	2021.11.29
PMR-65	GDDQ-2NQ-3	DeQing, Guangdong Province, China	2021.11.29
PMR-66	GDDQ-2NQ-4	DeQing, Guangdong Province, China	2021.11.29
PMR-67	GDDQ-2NQ-5	DeQing, Guangdong Province, China	2021.11.29
PMR-68	GDDQ-3NQ-1	DeQing, Guangdong Province, China	2021.11.29
PMR-69	GDDQ-3NQ-2	DeQing, Guangdong Province, China	2021.11.29
PMR-70	GDDQ-3NQ-3	DeQing, Guangdong Province, China	2021.11.29
PMR-71	GDDQ-3NQ-4	DeQing, Guangdong Province, China	2021.11.29
PMR-72	GDDQ-3NQ-5	DeQing, Guangdong Province, China	2021.11.29
PMR-73	GDDQ-4NQ-1	DeQing, Guangdong Province, China	2021.11.29
PMR-74	GDDQ-4NQ-2	DeQing, Guangdong Province, China	2021.11.29
PMR-75	GDDQ-4NQ-3	DeQing, Guangdong Province, China	2021.11.29
PMR-76	GDDQ-4NQ-4	DeQing, Guangdong Province, China	2021.11.29
PMR-77	GDDQ-4NQ-5	DeQing, Guangdong Province, China	2021.11.29
PMR-78	GDDQ-5NQ-1	DeQing, Guangdong Province, China	2021.11.29

PMR-79	GDDQ-5NQ-2	DeQing, Guangdong Province, China	2021.11.29
PMR-80	GDDQ-5NQ-3	DeQing, Guangdong Province, China	2021.11.29
PMR-81	GDDQ-5NQ-4	DeQing, Guangdong Province, China	2021.11.29
PMR-82	GDDQ-5NQ-5	DeQing, Guangdong Province, China	2021.11.29
PMR-83	GDDQ-6NQ-1	DeQing, Guangdong Province, China	2021.11.29
PMR-84	GDDQ-6NQ-2	DeQing, Guangdong Province, China	2021.11.29
PMR-85	GDDQ-6NQ-3	DeQing, Guangdong Province, China	2021.11.29
PMR-86	GDDQ-6NQ-4	DeQing, Guangdong Province, China	2021.11.29
PMR-87	GDDQ-6NQ-5	DeQing, Guangdong Province, China	2021.11.29
PMR-88	GDDQ-5NC-1	DeQing, Guangdong Province, China	2021.4.29
PMR-89	GDDQ-5NC-2	DeQing, Guangdong Province, China	2021.4.29
PMR-90	GDDQ-5NC-3	DeQing, Guangdong Province, China	2021.4.29
PMR-91	GDDQ-5NC-4	DeQing, Guangdong Province, China	2021.4.29
PMR-92	GDDQ-5NC-5	DeQing, Guangdong Province, China	2021.4.29
PMR-93	GDDQ-5NX-1	DeQing, Guangdong Province, China	2021.8.18
PMR-94	GDDQ-5NX-2	DeQing, Guangdong Province, China	2021.8.18
PMR-95	GDDQ-5NX-3	DeQing, Guangdong Province, China	2021.8.18
PMR-96	GDDQ-5NX-4	DeQing, Guangdong Province, China	2021.8.18
PMR-97	GDDQ-5NX-5	DeQing, Guangdong Province, China	2021.8.18
PMP-98	20211122-GSJN-S01	Gansu Guocao Pharmaceutical Co.	2021.11.22
PMP-99	20211122-GSJN-S02	Gansu Guocao Pharmaceutical Co.	2021.11.22
PMP-100	210901-1	JiuZhouTong HuangGang JinGui Factory	2021.9.1
PMP-101	210901-2	JiuZhouTong HuangGang JinGui Factory	2021.9.1
PMP-102	20211125-GSLM-S01	Gansu Longmai Herbs Co.	2021.11.25
PMP-103	20211125-NXMD-S01	Ningxia Mingde Chinese Medicine Co.	2021.11.25
PMP-104	20211125-NXMD-S02	Ningxia Mingde Chinese Medicine Co.	2021.11.25
PMP-105	20211129-JXZSTQT-S01	Jiangxi Zhangshu Traditional Chinese Medicine Pills Co.	2021.11.29
PMP-106	20211129-JXZSTQT-S02	Jiangxi Zhangshu Traditional Chinese Medicine Pills Co.	2021.11.29
PMP-107	20211129-JXZSTQT-S03	Jiangxi Zhangshu Traditional Chinese Medicine Pills Co.	2021.11.29
PMP-108	20211203-AGYPS01	Anguo Factory	2021.12.03
PMP-109	20211203-BZLDS01	Bozhou Lindan Chinese Medicine Company	2021.12.03
PMP-110	20211203-BZLDS02	Bozhou Lindan Chinese Medicine Co.	2021.12.03
PMP-111	20211222-AHWSS01	Anhui Wansheng Traditional Chinese Medicine Tablet Company	2021.12.22
PMP-112	20220104-ZJTJTS01	Zhejiang Tongjuntang Traditional Chinese Medicine Co.	2022.1.4
PMP-113	20220104-ZJTJTS02	Zhejiang Tongjuntang Traditional Chinese Medicine Co.	2022.1.4

Table S5. Sample collection information of PMRP.

Samples	Batch Number	Source	Processing Method	Collect Time
PRMP-01	AHBZSC202001Z	Chinese herbal medicine market	Unknown	2020.7.28
PRMP-02	AHBZSC202002Z	Chinese herbal medicine market	Unknown	2020.7.28
PRMP-03	AHBZSC202003Z	Chinese herbal medicine market	Unknown	2020.7.28
PRMP-04	AHBZSC202004Z	Chinese herbal medicine market	Unknown	2020.7.28
PRMP-05	AHBZSC202005Z	Chinese herbal medicine market	Unknown	2020.7.28
PRMP-06	SW01	Chinese herbal medicine market	Unknown	Unknown
PRMP-07	SW02	Chinese herbal medicine market	Unknown	Unknown
PRMP-08	SW03	Chinese herbal medicine market	Unknown	Unknown
PRMP-09	SW04	Chinese herbal medicine market	Unknown	Unknown
PRMP-10	SW05	Chinese herbal medicine market	Unknown	Unknown
PRMP-11	SW06	Chinese herbal medicine market	Unknown	Unknown
PRMP-12	GXYLSC202001Z	Chinese herbal medicine market	Unknown	2020.7.27

PRMP-13	SXXASC202001Z	Chinese herbal medicine market	Unknown	2020.7.27
PRMP-14	HBAGSC202001Z	Chinese herbal medicine market	Unknown	2020.7.27
PRMP-15	GDDQ	Chinese herbal medicine market	Unknown	Unknown
PRMP-16	AHWST01	Chinese herbal medicine market	Unknown	2021.11.22
PRMP-17	AHWST02	Chinese herbal medicine market	Unknown	2021.11.22
PRMP-18	AHWST03	Chinese herbal medicine market	Unknown	2021.11.22
PRMP-19	AHWST04	Chinese herbal medicine market	Unknown	2021.11.22
PRMP-20	AHWST05	Chinese herbal medicine market	Unknown	2021.11.22
PRMP-21	AHWST06	Chinese herbal medicine market	Unknown	2021.11.22
PRMP-22	AHWST07	Chinese herbal medicine market	Unknown	2021.11.22
PRMP-23	2015G0624	Chinese herbal medicine market	Unknown	Unknown
PRMP-24	2015G0057	Chinese herbal medicine market	Unknown	Unknown
PRMP-25	2015G0371	Chinese herbal medicine market	Unknown	Unknown
PRMP-26	2015G0251	Chinese herbal medicine market	Unknown	Unknown
PRMP-27	2015G0162	Chinese herbal medicine market	Unknown	Unknown
PRMP-28	2015G0239	Chinese herbal medicine market	Unknown	Unknown
PRMP-29	2015G0375	Chinese herbal medicine market	Unknown	Unknown
PRMP-30	2015G0625	Chinese herbal medicine market	Unknown	Unknown
PRMP-31	20211122-GSJN-Z01	Gansu Guocao Pharmaceutical Co.	Stewed for 4 h	2021.11.22
PRMP-32	20211122-GSJN-Z02	Gansu Guocao Pharmaceutical Co.	Stewed for 4 h	2021.11.22
PRMP-33	20211122-GSJN-Z03	Gansu Guocao Pharmaceutical Co.	Stewed for 8 h	2021.11.22
PRMP-34	20211122-GSJN-Z04	Gansu Guocao Pharmaceutical Co.	Stewed for 8 h	2021.11.22
PRMP-35	210901-ZHSW-01	JiuZhouTong HuangGang JinGui Factory	Steamed with black bean juice for 4 h	2021.9.1
PRMP-36	210901-ZHSW-02	JiuZhouTong HuangGang JinGui Factory	Steamed with black bean juice for 4 h	2021.9.1
PRMP-37	210901-ZHSW-03	JiuZhouTong HuangGang JinGui Factory	Steamed with black bean juice for 8 h	2021.9.1
PRMP-38	210901-ZHSW-04	JiuZhouTong HuangGang JinGui Factory	Steamed with black bean juice for 8 h	2021.9.1
PRMP-39	20211125-GSLM-Z01	Gansu Longmai Herbs Co.	Steamed with black bean juice for 5 h	2021.11.25
PRMP-40	20211125-GSLM-Z02	Gansu Longmai Herbs Co.	Steamed with black bean juice for 10 h	2021.11.25
PRMP-41	20211125-NXMD-Z01	Ningxia Mingde Chinese Medicine Co.	Steamed with black bean juice for 8 h	2021.11.25
PRMP-42	20211125-NXMD-Z02	Ningxia Mingde Chinese Medicine Co.	Steamed with black bean juice for 8 h	2021.11.25
PRMP-43	20211125-NXMD-Z03	Ningxia Mingde Chinese Medicine Co.	Steamed with black bean juice for 8 h	2021.11.25
PRMP-44	20211125-NXMD-Z04	Ningxia Mingde Chinese Medicine Co.	Steamed with black bean juice for 16 h	2021.11.25
PRMP-45	20211125-NXMD-Z05	Ningxia Mingde Chinese Medicine Co.	Steamed with black bean juice for 16 h	2021.11.25
PRMP-46	20211125-NXMD-Z06	Ningxia Mingde Chinese Medicine Co.	Steamed with black bean juice for 16 h	2021.11.25
PRMP-47	20211125-NXMD-Z07	Ningxia Mingde Chinese Medicine Co.	Steamed with black bean juice for 16 h	2021.11.25
PRMP-48	20211125-NXMD-Z08	Ningxia Mingde Chinese Medicine Co.	Steamed with black bean juice for 16 h	2021.11.25
PRMP-49	20211125-NXMD-Z09	Ningxia Mingde Chinese Medicine Beverage Co.	Steamed with black bean juice for 16 h	2021.11.25

PRMP-50	20211129-JXZSTQT-Z01	Jiangxi Zhangshu Traditional Chinese Medicine Pills Co.	Steamed with black bean juice for 4 h	2021.11.29
PRMP-51	20211129-JXZSTQT-Z02	Jiangxi Zhangshu Traditional Chinese Medicine Pills Co.	Steamed with black bean juice for 4 h	2021.11.29
PRMP-52	20211129-JZXSTQT-Z03	Jiangxi Zhangshu Traditional Chinese Medicine Pills Co.	Steamed with black bean juice for 4 h	2021.11.29
PRMP-53	20211129-JZXSTQT-Z04	Jiangxi Zhangshu Traditional Chinese Medicine Pills Co.	Steamed with black bean juice for 8 h	2021.11.29
PRMP-54	20211129-JZXSTQT-Z05	Jiangxi Zhangshu Traditional Chinese Medicine Pills Co.	Steamed with black bean juice for 8 h	2021.11.29
PRMP-55	20211129-JXZSTQT-Z06	Jiangxi Zhangshu Traditional Chinese Medicine Pills Co.	Steamed with black bean juice for 8 h	2021.11.29
PRMP-56	20211203-AGYPZ01	Anguo Factory	High-pressure steamed	2021.12.3
PRMP-57	20211203-BZLDZ01	Bozhou Lindan Chinese Medicine Co.	Steamed for 4 h-6 h	2021.12.3
PRMP-58	20211203-BZLDZ02	Bozhou Lindan Chinese Medicine Co.	Steamed for 4 h-6 h	2021.12.3
PRMP-59	20211222-AHWSZ01	Anhui Wansheng Traditional Chinese Medicine Tablet Company	Steamed for 2 h	2021.11.22
PRMP-60	20211222-AHWSZ02	Anhui Wansheng Traditional Chinese Medicine Tablet Company	Steamed for 2 h	2021.11.22
PRMP-61	20211222-AHWSZ03	Anhui Wansheng Traditional Chinese Medicine Tablet Company	Steamed for 3 h	2021.11.22
PRMP-62	20211222-AHWSZ04	Anhui Wansheng Traditional Chinese Medicine Tablet Company	Steamed for 3 h	2021.11.22
PRMP-63	20211222-AHWSZ05	Anhui Wansheng Traditional Chinese Medicine Tablet Company	Steamed for 4 h	2021.11.22
PRMP-64	20211222-AHWSZ06	Anhui Wansheng Traditional Chinese Medicine Tablet Company	Steamed for 4 h	2021.11.22
PRMP-65	20220104-ZJTJTZ01	Zhejiang Tongjuntang Traditional Chinese Medicine Co.	Steamed for 6 h	2022.4.1
PRMP-66	20220104-ZJTJTZ02	Zhejiang Tongjuntang Traditional Chinese Medicine Co.	Steamed for 6 h	2022.4.1
PRMP-67	20220104-ZJTJTZ03	Zhejiang Tongjuntang Traditional Chinese Medicine Co.	Steamed for 6 h	2022.4.1
PRMP-68	20220104-ZJTJTZ04	Zhejiang Tongjuntang Traditional Chinese Medicine Co.	Steamed for 6 h	2022.4.1
PRMP-69	20220104-ZJTJTZ05	Zhejiang Tongjuntang Traditional Chinese Medicine Co.	Steamed for 6 h	2022.4.1
PRMP-70	20220104-ZJTJTZ06	Zhejiang Tongjuntang Traditional Chinese Medicine Co.	Steamed for 6 h	2022.4.1
PRMP-71	20220104-ZJTJTZ07	Zhejiang Tongjuntang Traditional Chinese Medicine Co.	Steamed for 12 h	2022.4.1
PRMP-72	20220104-ZJTJTZ08	Zhejiang Tongjuntang Traditional Chinese Medicine Co.	Steamed for 12 h	2022.4.1
PRMP-73	20220104-ZJTJTZ09	Zhejiang Tongjuntang Traditional Chinese Medicine Co.	Steamed for 12 h	2022.4.1
PRMP-74	20220104-ZJTJTZ10	Zhejiang Tongjuntang Traditional Chinese Medicine Co.	Steamed for 12 h	2022.4.1
PRMP-75	20220104-ZJTJTZ11	Zhejiang Tongjuntang Traditional Chinese Medicine Co.	Steamed for 12 h	2022.4.1
PRMP-76	20220104-ZJTJTZ12	Zhejiang Tongjuntang Traditional Chinese Medicine Co.	Steamed for 12 h	2022.4.1
